# 3D-Printed Double-Helical Biodegradable Iron Suture Anchor: A Rabbit Rotator Cuff Tear Model

**DOI:** 10.3390/ma15082801

**Published:** 2022-04-11

**Authors:** Wen-Chih Liu, Chih-Hau Chang, Chung-Hwan Chen, Chun-Kuan Lu, Chun-Hsien Ma, Shin-I Huang, Wei-Lun Fan, Hsin-Hsin Shen, Pei-I Tsai, Kuo-Yi Yang, Yin-Chih Fu

**Affiliations:** 1 Ph.D. Program in Biomedical Engineering, College of Medicine, Kaohsiung Medical University, Kaohsiung 80756, Taiwan; andysirliu@gmail.com (W.-C.L.); hwan@kmu.edu.tw (C.-H.C.); 2 Department Orthopedics, Kaohsiung Medical University Hospital, Kaohsiung Medical University, Kaohsiung 80756, Taiwan; 3 Regeneration Medicine and Cell Therapy Research Center, Kaohsiung Medical University, Kaohsiung 80756, Taiwan; 4 Orthopedic Research Center, Kaohsiung Medical University, Kaohsiung 80708, Taiwan; 5 Division of Plastic Surgery, Department of Surgery, Kaohsiung Medical University Hospital, Kaohsiung Medical University, Kaohsiung 80756, Taiwan; igor8301023@gmail.com; 6 Graduate Institute of Animal Vaccine Technology, College of Veterinary Medicine, National Pingtung University of Science and Technology, Pingtung 912301, Taiwan; 7 Department of Orthopedic Surgery, Kaohsiung Municipal Ta-Tung Hospital, Kaohsiung 80145, Taiwan; 8 Department of Healthcare Administration and Medical Informatics, Kaohsiung Medical University, Kaohsiung 80708, Taiwan; 9 Department of Orthopedics, College of Medicine, Kaohsiung Medical University, Kaohsiung 80708, Taiwan; 10 Institute of Medical Science and Technology, National Sun Yat-sen University, Kaohsiung 80420, Taiwan; 11 Department of Orthopedic Surgery, Park One International Hospital, Kaohsiung 81367, Taiwan; u9001054@yahoo.com.tw; 12 Biomedical Technology and Device Research Laboratories, Industrial Technology Research Institute, Hsinchu 31057, Taiwan; chm0406@itri.org.tw (C.-H.M.); sophiashini@itri.org.tw (S.-I.H.); wlfan@itri.org.tw (W.-L.F.); shenhsin@itri.org.tw (H.-H.S.); peiyi@itri.org.tw (P.-I.T.)

**Keywords:** 3D printing, biodegradable metal, iron, suture anchor, rabbit, rotator cuff

## Abstract

Suture anchors are extensively used in rotator cuff tear surgery. With the advancement of three-dimensional printing technology, biodegradable metal has been developed for orthopedic applications. This study adopted three-dimensional-printed biodegradable Fe suture anchors with double-helical threads and commercialized non-vented screw-type Ti suture anchors with a tapered tip in the experimental and control groups, respectively. The in vitro study showed that the Fe and Ti suture anchors exhibited a similar ultimate failure load in 20-pound-per-cubic-foot polyurethane foam blocks and rabbit bone. In static immersion tests, the corrosion rate of Fe suture anchors was 0.049 ± 0.002 mm/year. The in vivo study was performed on New Zealand white rabbits and SAs were employed to reattach the ruptured supraspinatus tendon. The in vivo ultimate failure load of the Fe suture anchors was superior to that of the Ti suture anchors at 6 weeks. Micro-computed tomography showed that the bone volume fraction and bone surface density in the Fe suture anchors group 2 and 6 weeks after surgery were superior, and the histology confirmed that the increased bone volume around the anchor was attributable to mineralized osteocytes. The three-dimensional-printed Fe suture anchors outperformed the currently used Ti suture anchors.

## 1. Introduction

Rotator cuff repair is one of the most common surgeries in the upper extremities [1]. The current trend in rotator cuff repairs is the use of suture anchors (SAs), such as metallic SAs and nondegradable or biodegradable polymer SAs [2,3]. The two most commonly used metal SAs are stainless steel and titanium SAs [4]. Stainless steel SAs are encapsulated by an inflammatory cell-rich fibrous membrane, which impedes the osteoblasts from binding to the surface [4], whereas Ti forms a layer of Ca and phosphate, which facilitate osteointegration [5]. Although metallic SAs provide a rigid fixation and have been used for a long time, they have the disadvantage of permanent metal artifact interference with diagnostic imaging [6]. Biodegradable SAs, such as poly-L-lactic acid, is radiolucent; however, inflammatory reactions can develop, leading to osteolysis [7], interosseous cyst formation, and the bone not being replaced after the implants degrade [2]. These complications have led to the use of biologically inert and nondegradable materials, such as polyether ether ketone (PEEK), as popular biomaterials for use in SAs [8].

Biodegradable metallics are promising biomaterials due to their combination of a temporarily high mechanical load-bearing capacity and gradual degradation [9,10]. Three metals, namely, Mg [11,12], Fe [13], and Zn [14], have been developed as biodegradable implants [15]. Magnesium-based implants have been the focus of biodegradable metal implants and extensive data from basic to translational research is available [16,17,18]. The drawback of Mg-based implants is their fast biodegradation and generation of H gas [19]. Iron-based implants exhibit a slow in vivo degradation rate as compared to Mg-based implants [20]. Zinc-based alloys were introduced more recently, and the corrosion rate falls between that of Mg- and Fe-based biodegradable metals [21]. Pure Zn has relatively poor strength; however, Zn and its alloys are favorable materials for biodegradable implants for osteosynthesis [22]. Fe-based biodegradable materials have been demonstrated to have similar mechanical properties to natural bone and satisfactory cytocompatibility [20,23,24]. To overcome the slow degradation rate of Fe-based implants, porous-based implants fabricated with additive manufacturing technology have been used for Fe-based SAs, showing satisfactory in vivo cytotoxicity and cytocompatibility, biological performance, and biomechanical properties as compared to commercial biodegradable SAs in our previous rabbit shinbone study [25]. Considering the corrosion rate, biocompatibility, and mechanical strength, Fe-based materials for orthopedic applications have been vigorously developed [26].

Even with the advancements in biodegradable metal materials, there is still a scarcity of Fe-based biodegradable SA data in an animal model. The rotator cuff muscle architecture of rabbits is more similar to that of humans than any other large mammal, such as dogs, sheep, pigs, and cows [27]. The novelty of this study is that we adopted a rabbit model, creating a supraspinatus tendon (SST) tear and repairing the SST tear with two kinds of SA. The experimental group comprised a three-dimensional (3D)-printed 3.5 mm open-construct coil-type biodegradable Fe SA. It is easier to manufacture helical structures with high porosity using 3D printing technology. To overcome the slow degradation property of Fe, we designed the open architecture of the SA to increase the surface area and accelerate the corrosion rate. In addition, the double-helical and high-porosity architecture provides better structural stiffness and bone osteointegration with the SA. The control group comprised a 3.5 mm non-vented screw-type Ti SA [28]. The aim of this study was to evaluate the in vitro and in vivo biomechanical performance, micro-computed tomography (micro-CT) results, and histopathological analysis findings of Fe SAs and compare them with those of the Ti SA by using a rabbit SST model. The objective of this study was to compare the biomechanical, radiographic, and histopathological performance of 3D-printed open-construct double-helical biodegradable Fe SA and Ti SA as prospective SAs in osteointegration.

## 2. Materials and Methods

### 2.1. Production and In Vitro Tests of Double-Helical Biodegradable Fe SAs

**Figure 1:**
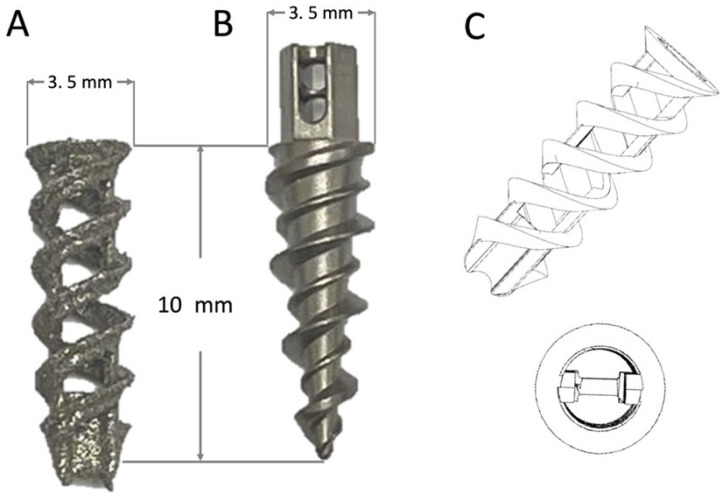
Specifications of the (**A**) open-construct double-helical Fe suture anchor (SA) and (**B**) screw-type Ti SA. (**C**) Illustration of the open-construct double-helical Fe SA.

The open-construct coil-type Fe SA was produced using additive-manufactured selective laser sintering (SLM) technology (SLM EOSINT M 270 model; EOS GambH-Electro Optical Systems, Krailling, Germany). The 3.5 mm open-construct SAs were designed to have a circular cross-section with a double-helical threaded cylindrical shape to increase the surface area (Figure 1A,C). Its tip had a crossbar for the suture to loop over it. The SAs were prepared using biodegradable spherical Fe powder with a purity of >99.5%. For comparison, screw-type titanium SAs (TWINFIX Ti 3.5 mm Suture Anchor, Smith & Nephew, London, UK) were used as the controls (Figure 1B).

**Figure 2:**
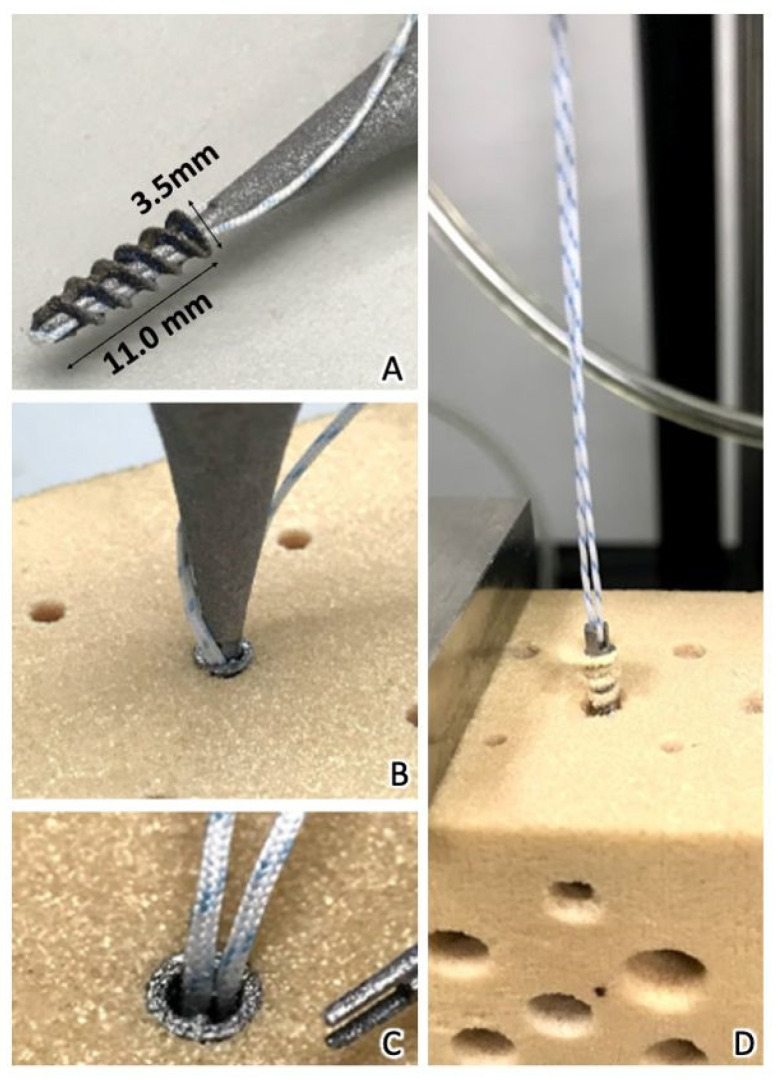
(**A**) The dimensions of the Fe SA were 11.0 × 3.5 mm. (**B**) An inserter handle was designed to hold the Fe SA, which was inserted into a predrilled hole on the polyurethane foam block. (**C**) After inserting the SA and removing the inserter handle, the suture exited the core of the iron SA. (**D**) The control group: the non-vented screw-type Ti SA had a tapered tip portion with sutures through the proximal eyelet. The suture was passed through the eyelet at the top of the SA. The Ti SA was pulled out of the polyurethane foam block during the mechanical test.

In vitro mechanical tests were conducted to evaluate the mechanical characteristics of the SAs. The tests were performed using a 20-pound-per-cubic-foot (pcf) polyurethane foam block (part# 1522-03; Sawbone, Pacific Research Laboratories, Vashon, WA, USA). A No. 2 ultra-high molecular weight polyethylene fiber suture (Ultrabraid, Smith & Nephew, London, UK) with equal limbs was threaded through the suture eyelet, looped, and fixed over a post on the adapter before mechanical testing (Figure 2). The static ultimate pullout strength was determined at a displacement rate of 1 mm/s. The mechanical tests were performed using an Instron E3000 (ElectroPuls, Instron, MA, USA).

### 2.2. Corrosion Rate of Pure Fe SAs Using Static Immersion Tests

Static immersion tests were performed to evaluate the weight loss of the Fe SA. Six samples were weighed to obtain their initial weights (g) and surface areas (mm^2^). The experimental specimens were immersed in 10 mL of Hanks’ solution for 3 months and the temperature was maintained at 37 °C using a heating mantle. The samples were removed from the solution after every 30 days of treatment, ultrasonically cleaned, air dried, and weighed. The mass changes were used to calculate the corrosion rate (mm/year) using Equation (1), which is based on the ASTM G31 standard [29]:

CR = kΔm/(ρAt)(1)

where CR is the corrosion rate, K is a constant (8.76 × 10^4^), Δm (g) is the weight loss of the sample, ρ (g/cm^3^) is the density of the object, A (cm^2^) is the initial surface area of the sample, and t (h) is the immersion time of the sample in the solution.

### 2.3. In Vivo Animal Study Design

All animal experiments were approved by the Ethics Committee of the Biomedical Technology and Device Research Laboratories of the Industrial Technology Research Institute in accordance with national animal welfare legislation (approval no.: ITRI-IACUC-2020-050), and the study protocol conformed to the National Institute of Health guidelines for the use of laboratory animals. A total of 24 New Zealand white rabbits (Animal Health Research Institute of the Council of Agriculture) with a mean body weight of 4.0 ± 0.4 kg at the age of 6 months were selected. Each rabbit shoulder joint was randomized into experimental and control groups by using a computer-generated randomization method. In the control group, Ti SAs were implanted in the greater tuberosity of the humerus. In the experimental group, Fe SAs were implanted using the same surgical procedure as that in the control group. Four rabbits were euthanized immediately after SA implantation and frozen for biomechanical testing. The other rabbits were further divided into two subcategories based on implantation periods of 2 weeks and 6 weeks after surgery (10 rabbits in each group). Micro-CT and biochemical tests were performed 2 and 6 weeks after the surgery. Subsequently, the specimens were frozen for use in further biomechanical tests.

### 2.4. Surgical Methods

All surgical procedures were performed under general anesthesia by administering an intramuscular injection of a Zoletil–Rompun mixture (Zoletil 15 mg/kg; Rompun 0.05 mL/kg; Zoletil, Virbac Taiwan, Taipei, Taiwan; Rompun, Bayer Taiwan, Taipei, Taiwan). To induce analgesia, the rabbits were given intramuscular ketoprofen (2 mg/kg, ASTAR, Hsinchu, Taiwan) 24 h preoperation, and for 7 consecutive days following the surgery. For the prophylaxis of infection, the rabbits were given intramuscular Gentamycin (5 mg/kg, Standard Chem. & Pharm, Tainan, Taiwan) 24 h preoperation and for 7 consecutive days following the surgery.

**Figure 3:**
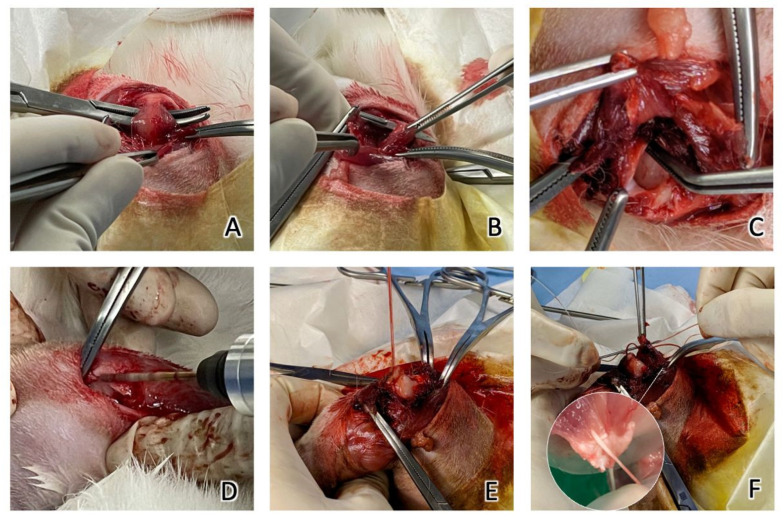
(**A**) The SST of the rabbit was identified. (**B**) The SST was sharply dissected with a scalpel blade and (**C**) the SST was detached from the insertion of the humerus. (**D**) A drill bit was used to predrill a hole in the humerus insertion of the SST. (**E**) After inserting the SA, the two ends of the suture exited from the SST insertion. (**F**) The SST was repositioned to the footprint using a modified Mason–Allen stitch.

**Figure 4:**
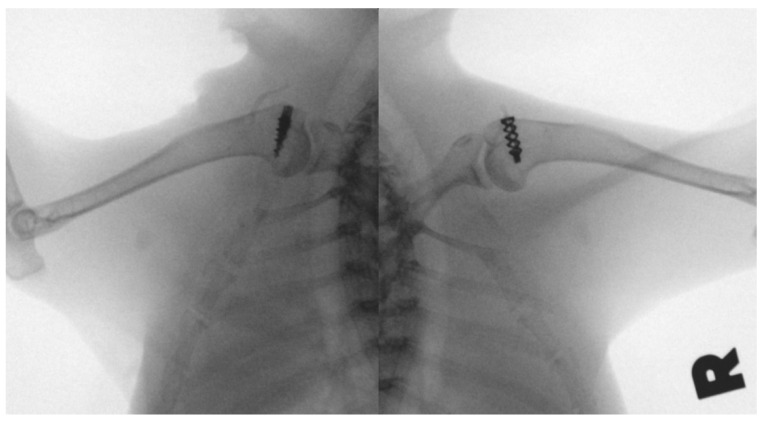
The radiograph shows that the Ti SA (left shoulder) and Fe SA (right shoulder) were inserted into the proximal humerus greater tuberosity.

Surgical procedures were performed following the method reported by Louati et al. with some modifications [30]. The supraspinatus insertion was sharply detached from the greater tuberosity, simulating a complete tear. A scalpel blade was used to decorticate the SSP footprint. A hole was predrilled on the lateral and distal to the footprint in the cortical bone with a 1.5 mm drill bit. In the experimental group, Fe SAs with No. 2 Ultrabraid sutures were inserted into the predrilled hole. The tendon was repositioned to the footprint using a modified Mason–Allen stitch. In the control group, Ti SAs with No. 2 Ultrabraid sutures were used. The tendon was repositioned to the footprint with the same technique as the experimental group (Figure 3). The deltoid was closed, followed by skin closure. The position of the SAs was confirmed postoperatively using X-ray imaging (Figure 4). All animals were euthanized after the experiments were completed by administering an intravenous overdose of pentobarbital.

### 2.5. Micro-CT Analysis

After the rabbits were euthanized, 10 specimens were retrieved from each group, and multi-scale nano-CT (Skyscan 2211, Bruker Micro-CT, Kontich, Belgium) was used for 30 μm voxel resolution. A voltage of 155 kVp, an 80 μA current, and a 6 W output in micro-focus mode with a 360° scan was used for the Ti implants. A voltage of 180 kVp, a 100 μA current, and an 18 W output in high-power mode with a 360° scan was used for the Fe implants.

InstaRecon xCBR (version 2.0.4.6, InstaRecon, Champaign, IL, USA) and NRecon (Bruker Micro-CT, Kontich, Belgium) were used for image reconstruction. Finally, NRecon was used for ring artifact and beam hardening correction.

**Figure 5:**
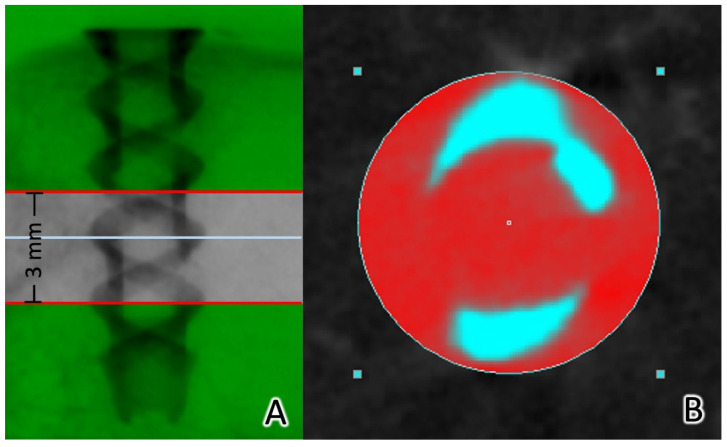
The reconstructed cross-sections were re-orientated, and the region of interest (ROI) was further selected. The 3.5 mm implant column was isolated. (**A**) The analysis was performed with 3 mm images (100 slices, 3–6 mm from the end of the implant). Automatic Ostu thresholding and bone ingrowth analysis were performed using CTAn software. (**B**) The ROI was defined as a 200–1000 μm region around the implant.

To reposition the reconstructed cross-section and select the region of interest (ROI), the 3.5 mm implant column was isolated. Three mm (100 slices) images were used for the analysis. CTAn software was used for automatic Ostu thresholding and bone growth analysis. A 200–1000 μm region around the implant was defined as the ROI for bone growth analysis (Figure 5). The bone and metal structures could be separated according to the differences in X-ray absorption. CTAn software with a shrink-wrap algorithm was employed to identify the border of the metallic structure. The tissue volume (TV, mm^3^), bone volume (BV, mm^3^), and bone surface area (BS, mm^2^) were measured for the 200–1000 μm ROI surrounding the metallic implant in the bone. To determine the BV percentage, the BV/TV ratio (BV/TS, %) was calculated. The BS area per total volume (BS/TV, 1/mm) in the 3D analysis was used for characterizing the bone to implant contact. Subsequently, a “sphere-fitting” measurement method was used to analyze the entire object volume (OV, mm^3^), object surface (OS, mm^2^), and object thickness (OT, mm) [31,32,33]. Bone formation and implant degradation curve analyses were performed. Avizo software (Thermo Fisher Scientific, MA, USA) and CTVox (Bruker Micro-CT, Kontich, Belgium) were used for the 3D visualization.

### 2.6. Biomechanical Analysis

Two rabbits were euthanized immediately postimplantation, and ten rabbits (20 shoulders) were euthanized 2 and 6 weeks postoperation. Ten shoulders were retrieved and used for biomechanical analyses to investigate the failure load and the site of failure [30], which could explain the in vivo failure load and failure site of the repairs of SST using SAs. The SST and proximal humerus were isolated to ensure that only the SST attached to the humerus head contributed to the mechanical evaluation. The biomechanical testing was performed at room temperature (25 °C). After removing the redundant tissue, the humerus was embedded into a custom-made metallic clamp at the bottom of the testing machine (Instron E3343; ElectroPuls, Instron, MA, USA). The tendons were positioned along their anatomic direction of pull, at an angle of 45° to the longitudinal axis of the humeral shaft. This was followed by tensile loading to failure at 1 mm/s, where a 50% drop in tensile strength was defined as the breaking point. The load and displacement data were collected, and the mode of failure was noted. The load at failure was determined using Bluehill LE v3.71.4609 (Instron, Norwood, MA, USA). The failure modes were defined as failure at the tendon–suture junction, failure at the suture–anchor junction, and SA pullout.

### 2.7. Histological Analysis

Ten specimens were retrieved from each group for histological analysis. All the harvested samples were fixed in 10% formalin for 14 days and sequentially dehydrated with increasing concentrations of ethanol (70, 95, and 100%) for at least 1 d and infiltrated for 5 d by using polymethylmethacrylate [34,35]. After embedding, the samples were cut vertically, perpendicular to the long axis of the SA, at the level of the bone–implant interfaces. The sections were cut to approximately 150 μm in thickness by using a low-speed saw (IsoMet, Buehler, Lake Bluff, IL, USA) and ground to 60 μm by using a grinding and polishing machine [36]. The ground sections were stained with Sanderson’s rapid bone stain (Dorn & Hart Microedge Inc., Loxley, AL, USA) and then counterstained with acid fuchsin. All bone–implant interfaces were examined using a light microscope (Nikon Eclipse Ti-series, Melville, NY, USA).

### 2.8. Biochemical Analysis

Blood samples were obtained before surgery and 2 weeks and 6 weeks after surgery. The blood serum was processed in an ISO 15189:2012 [37] accreditation of medical laboratories using an automated spectrophotometer (ADVIA Chemistry XPT System, Siemens Healthineers, Germany) for analysis of the following parameters: blood urea nitrogen (BUN), creatinine (Cr), alanine transaminase (ALT), and albumin (Alb).

### 2.9. Statistical Analysis

All experimental data are presented as the mean ± standard deviation. The Wilcoxon rank-sum test was used for the nonparametric analysis. A *p*-value of <0.05 was considered statistically significant. Statistical analysis was performed using SPSS statistics (version 26, Chicago, IL, USA).

## 3. Results

### 3.1. In Vitro Mechanical Analyses of the Bioabsorbable Fe SA

**Figure 6:**
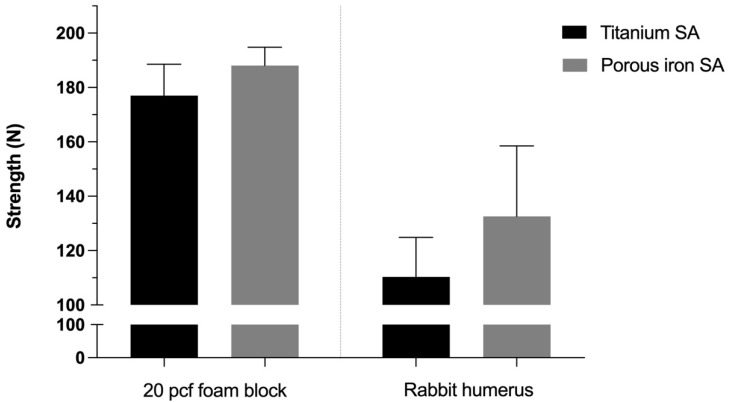
In vitro biomechanical ultimate pullout strength assessment for the different SAs in 20-pound-per-cubic-foot (pcf) polyurethane foam blocks and rabbit humeri. Mean ± standard error of the mean (SEM).

The ultimate in vitro pullout strength of the Fe SA (188.03 ± 13.56 N) was similar to that of the Ti SA (177.00 ± 20.04 N, *p* = 0.157) in 20 pcf polyurethane foam blocks. The ultimate pullout strength of the Fe SA (132.55 ± 44.92 N) was similar to that of the Ti SA (110.35 ± 25.09 N, *p* = 0.827) in the rabbit humeri (Figure 6).

### 3.2. Static Immersion Test

**Figure 7:**
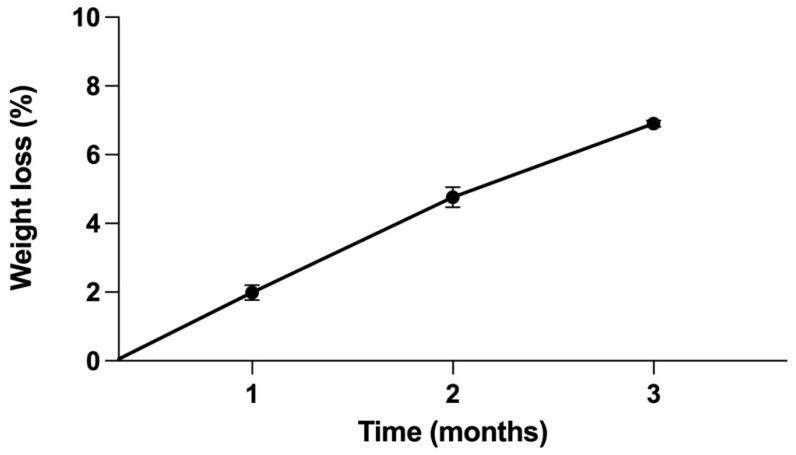
In vitro corrosion characteristics of the Fe SA. Mean ± SEM.

The mean weight loss at 30, 60, and 90 d were 1.99 ± 0.52, 4.76 ± 0.72, and 6.91 ± 0.22% respectively. The corrosion rate was 0.049 ± 0.002 mm/year (Figure 7).

### 3.3. In Vivo Biomechanical Analysis

**Figure 8:**
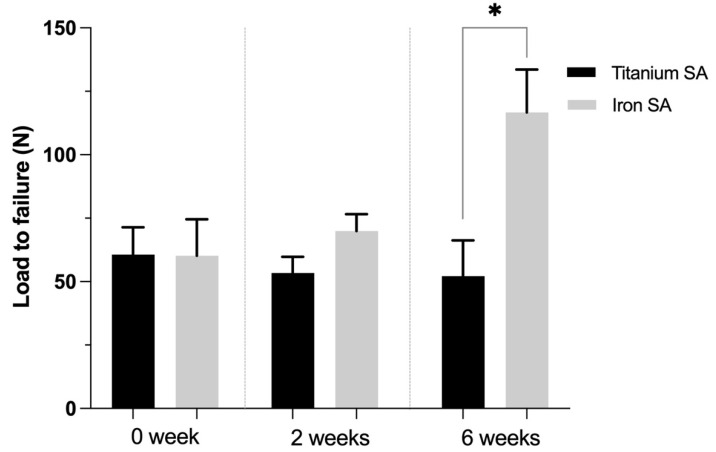
In vivo biomechanical ultimate pullout strength assessment for different SAs at 0, 2, and 6 weeks after surgery. Mean ± SEM. * *p* < 0.05.

The biomechanical analysis results revealed no difference between the ultimate failure load of the Fe SA and that of the Ti SA (60.60 ± 15.28 N, *p* = 1.000) at 0 weeks (60.22 ± 28.73 N). At 2 weeks, there was no significant difference between the ultimate failure load of the Fe SA and Ti SA (69.94 ± 16.18 N and 53.36 ± 15.74 N, *p* = 0.093, respectively). At 6 weeks, there was a significant difference between the ultimate failure load of the Fe SA and Ti SA (116.64 ± 33.80 N and 52.14 ± 28.20 N, *p* = 0.043, respectively) (Figure 8).

### 3.4. Failure Sites

**Figure 9:**
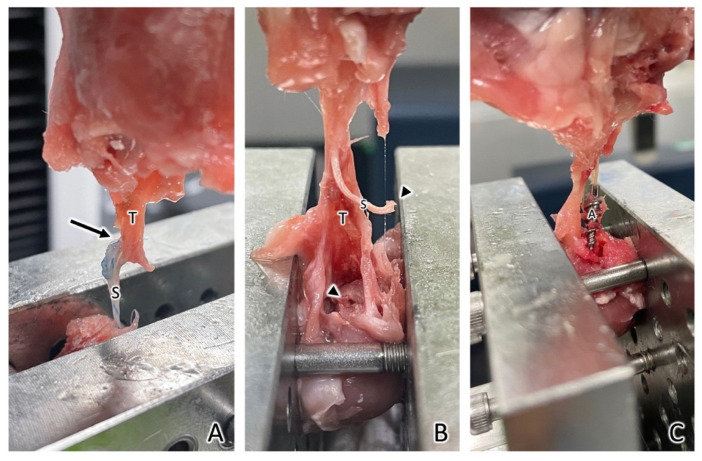
Three different modes of failure after the ultimate pullout strength assessment. (**A**) Failure at the tendon–suture junction (arrow). (**B**) Failure at the suture–anchor junction. That is, the end of the suture ruptured from the anchor (arrowhead). (**C**) The SA was pulled out. T, tendon; S, suture; A, anchor.

At week 0, all repaired tendons failed at the tendon–suture junction in both the Fe and Ti SA groups. At week 2, there were five out of six (83.3%) failures at the tendon-suture junction, one out of six (16.7%) failures at the suture–anchor junction, and no SA was pulled out in the Fe SA group. In the control group, there were three out of six (50%) failures at the tendon–suture junction, two out of six (33.3%) failures at the suture–anchor junction, and one SA (16.7%) was pulled out from the bone. At week 6, there were four out of six (66.7%) failures at the tendon–suture junction (Figure 9A), two out of six (33.3%) failures at the suture–anchor junction, and no SA was pulled out in the Fe SA group (Figure 9B). In the control group, there were five out of six (83.3%) failures at the tendon–suture junction, and one SA (16.7%) was pulled out of the bone (Figure 9C).

### 3.5. Micro-CT Analysis

**Figure 10:**
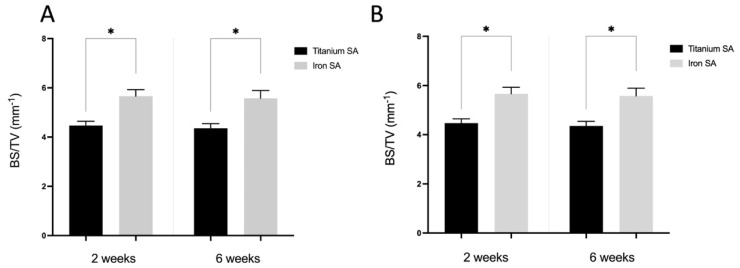
Micro-computed tomography (micro-CT) analysis. Quantitative evaluation of the bone volume (BV) between the bone and SAs. The tissue volume (TV, mm^3^), BV (mm^3^), and BS (mm^2^) were examined in a region of interest (ROI) of 200–1000 μm around the implant. (**A**) BV fraction (BV/TV, %) and (**B**) BS density (BS/TV, mm^−1^) represent the BV rate and bone tissue surface rate, respectively. Mean ± SEM. * *p* < 0.05.

**Figure 11:**
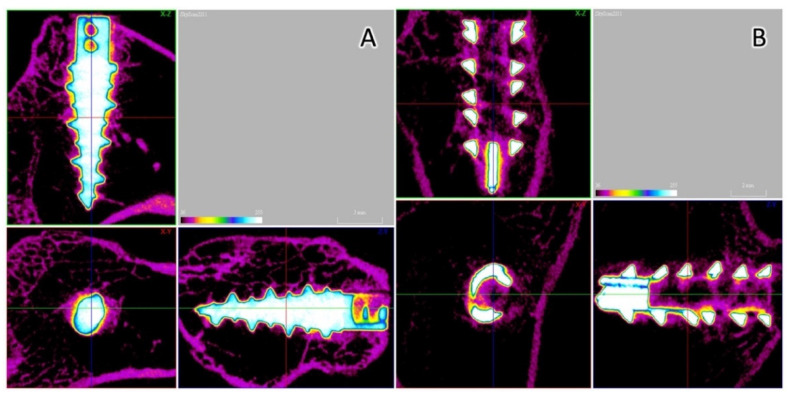
Micro-CT analysis. (**A**) Titanium SA 2 weeks after implantation. The BV fraction was 27.77% and the bone surface (BS) density was 4.52 mm^−1^. (**B**) Iron SA 2 weeks after implantation. The BV fraction was 38.20% and the BS density was 6.05 mm^−1^.

**Figure 12:**
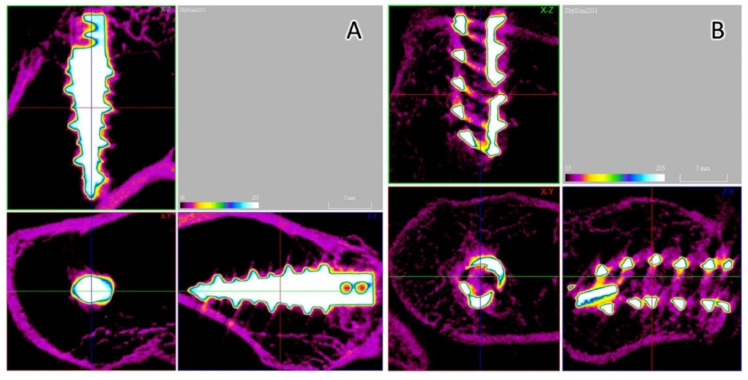
Micro-CT analysis. (**A**) Ti SA 6 weeks after implantation. The BV fraction was 29.59% and the BS density was 4.57 mm^−1^. (**B**) Fe SA 6 weeks after implantation. The BV fraction was 39.78% and the BS density was 6.31 mm^−1^.

Micro-CT was performed to evaluate the bone formation between the implant and bone tissue. The Fe SA exhibited a higher postoperative BV/TV at 2 weeks (35.84 ± 3.80 vs. 27.18 ± 4.46, *p* = 0.003) and 6 weeks (33.47 ± 3.78 vs. 27.46 ± 2.14, *p* = 0.001) as compared to the Ti SA (Figure 10A). The Fe SA exhibited a higher postoperative BS/TV at 2 weeks (5.66 ± 0.76 vs. 4.47 ± 0.53, *p* = 0.005) and 6 weeks (5.58 ± 0.89 vs. 4.36 ± 0.56, *p* = 0.005) as compared to the Ti SA (Figure 10B). There was no difference in the BV/TV intra-group analysis between the 2- and 6-week samples in the Ti SA group (*p* = 0.453) and in the Fe group SA (*p* = 0.294). There was no difference in the BS/TV intra-group analysis between the 2- and 6-week samples in the Ti SA group (*p* = 0.860) and in the Fe group SA (*p* = 0.793). Figure 11 and Figure 12 show examples of the Ti SA and Fe SA at 2 and 6 weeks, respectively, after the SA implantation.

**Figure 13:**
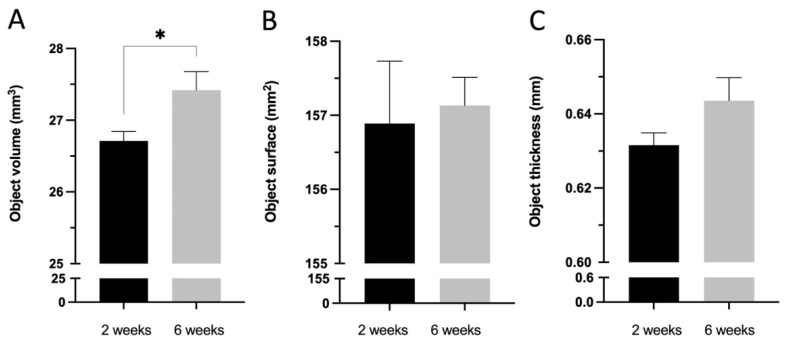
Micro-CT degradation analysis of the Fe SA groups 2 and 6 weeks postoperation in (**A**) Objective volume (mm^2^), (**B**) Object surface (mm^2^), and (**C**) Object thickness (mm). Mean ± SEM. * *p* < 0.05.

**Figure 14:**
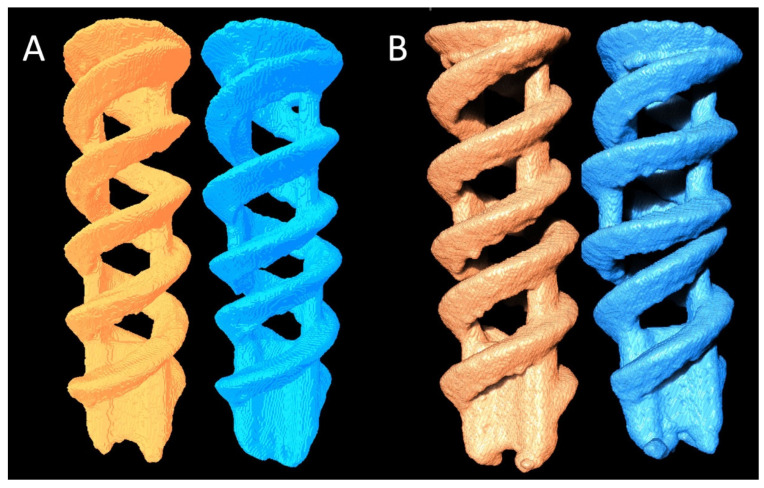
Reconstructed micro-CT images of two Fe SAs (**A**) 2 weeks postoperation and (**B**) 6 weeks postoperation.

The Fe SA degradation analysis showed an OV increase at 6 weeks as compared to that at 2 weeks (27.42 ± 0.81 vs. 26.71 ± 0.41, *p* = 0.021); however, the OS and OT at 2 and 6 weeks exhibited no significant difference (157.13 ± 1.20 vs. 156.89 ± 2.66, *p* = 0.173 and 0.64 ± 0.02 vs. 0.63 ± 0.01, *p* = 0.240, respectively), as shown in Figure 13. Figure 14 shows the reconstructed micro-CT images of the Fe SA at 2 and 6 weeks.

### 3.6. Histological Analyses

**Figure 15:**
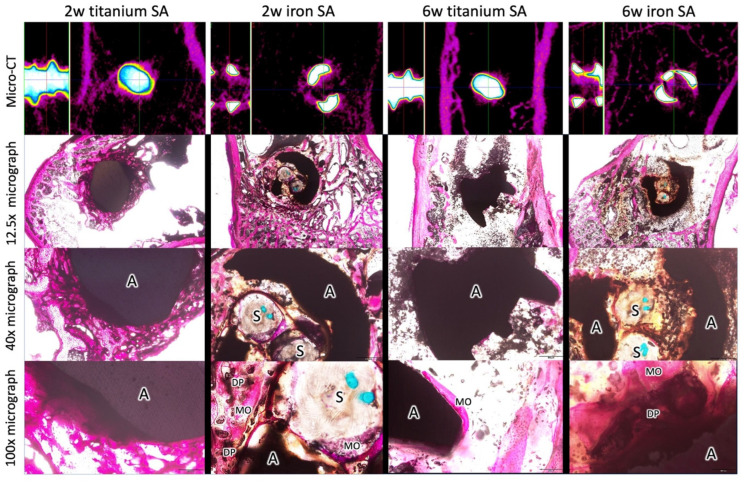
Micro-CT and histological examination of the bone–SA interface 2 and 6 weeks postoperation. w, week; A, anchor; S, suture; DP, degradation products; MO, mineralized osteocytes.

The mineralized bone formation was observed in the Ti SA and Fe SA groups at 2 and 6 weeks postoperation (Figure 15). In all the histological analyses, mineralized osteocytes were observed in the region that closely contacted the SA. At 2 weeks, new bone formation was observed around the Fe SA, which penetrated the suture fiber. More degradation products were observed surrounding the FE SA at 6 weeks as compared to that of the Fe SA at 2 weeks.

### 3.7. Biochemical Analysis

**Figure 16:**
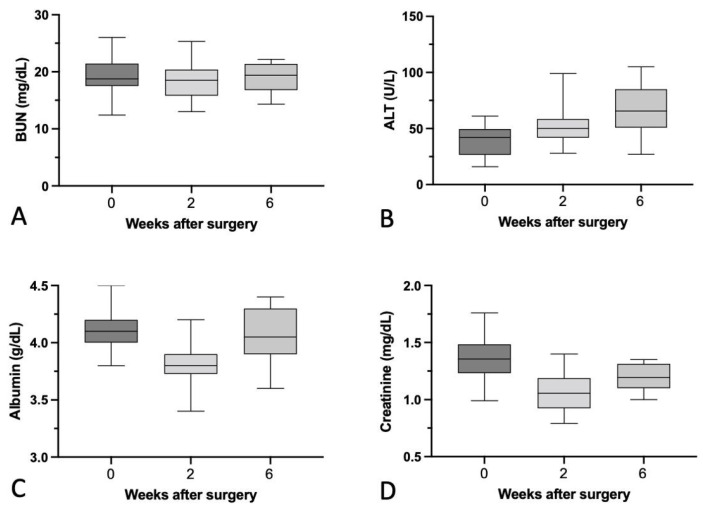
(**A**) Level of serum blood urea nitrogen (BUN; mg/dL), (**B**) level of serum alanine transaminase (ALT; U/L), (**C**) level of serum albumin (Alb; g/dL), and (**D**) level of creatinine (Cr; mg/dL) preoperation and 2 and 6 weeks postoperation. Mean ± SEM.

Blood samples were collected from all the rabbits preoperation and at 2 and 6 weeks postoperation for biochemical analysis. Table 1 and Figure 16 show that the serum ALT level was increased gradually at 2 and 6 weeks postoperation. However, the serum Cr, Alb, and BUN levels were comparable at both time points.

## 4. Discussion

The results showed that the in vitro biomechanical data of the 3D-printed Fe SA was similar to that of the Ti SA (Figure 6). The corrosion rate of the Fe SA was 0.049 ± 0.002 mm/year (Figure 7). The in vivo data in the rabbit model demonstrated that the Fe SA exhibited a higher pullout strength and showed more mineralized bone formation 2 and 6 weeks postoperation as compared to the Ti SA (Figure 8).

Corrosion behavior is one of the most important factors for biodegradable materials. The optimal value of corrosion rate for plates and screws is approximately 0.5 mm/year [14]. If the corrosion rate is too high, the mechanical strength will deteriorate before healing, leading to implant failure. If the corrosion rate is too low, delays in the healing of the bone tissue might cause adverse effects. The in vitro corrosion rates vary with either the static immersion test or polarization test using different physiological solutions. The Fe SA employed in this study was produced using SLM technology and Fe powder with an Fe purity of > 99.5%. The corrosion rate calculated using the 3-month static immersion test was 0.049 ± 0.002 mm/year, which was similar to scaffolds produced using extrusion-based 3D printing with an Fe power purity of 99.88% (0.05 mm/year) [13], but was slower than that of pure Fe with a refined structure produced using electroforming (0.40 mm/year) [38], soft ingot Fe (>99.8% purity) in the form of 2 mm thick sheets produced using cross-rolling (0.11–0.14 mm/year) [39], and composites produced using SLM with C nanotubes/Fe (99% purity) (0.085 mm/year) [40]. Overall, pure Fe’s corrosion rate ranges from 0.049 to 0.4 mm/year. In comparison to biodegradable Fe, the in vitro corrosion rate of pure Mg ranges from 0.33 to 0.99 mm/year [17,41,42], and that of pure Zn ranges from 0.014 to 0.75 mm/year [43,44,45]. The in vitro corrosion rate of pure Fe is slower than that of pure Mg and Zn.

The architecture of small mammal rotator cuffs shows greater similarity to that of humans than that of large mammals [27]. Some rabbit rotator cuff tear studies have been proposed to demonstrate the outcomes of repairing these using SAs [30,46]. Chaler et al. [46] demonstrated that repairing SST ruptures using SAs yielded higher loads to failure immediately after the repair and after the first postoperative week as compared to the transosseous repair. Louati et al. [30] demonstrated the outcomes of the channeling and no-channeling techniques in the repair of SST ruptures using SAs. Both animal studies showed no difference in the load to failure after 4 weeks of SA implantation [30,46]. Consequently, the authors hypothesized that 6 weeks after SA implantation is a long enough period to examine the ultimate load to failure. The in vivo biomechanical analysis mimicked the supraspinatus muscle pullout direction. The biomechanical results showed the most common failure site was at the tendon–suture junction, implying that the tendon was cut by the sutures (Figure 9). Although the load to failure was similar between the two groups, there was no SA pullout failure in the Fe SA group but there was one SA pullout failure in the Ti SA group.

Micro-CT was used to quantify the BV fraction (BV/TV) and BS density (BS/TV) of the ROI surrounding the SA [47]. A higher BV/TV is better for bone growth and a higher BS/TV indicates an increased bone growth closer to the implant surface region. The results of this study demonstrate that the Fe SA yielded increased bone growth as compared to the Ti SA, especially in the region near the implant surface. This result corresponded to the histology results, where the BV, identified using micro-CT, was mineralized bone. Due to the open-construct design of the Fe SA, new mineralized bone growth into the core of the anchor occurred as early as week 2 (Figure 14). The micrograph demonstrated new bone grew into the Fe SA and embedded the sutures, which may provide stability, even if degradation occurred in the Fe SA (Figure 15). In our previous study, the cell sensitivity assays showed that the Fe SA exhibited no cytotoxicity and lamellipodial extrusions from the cells, and the attachment on the implant surface could be identified using a scanning electron microscope [25]. Additionally, no significant Fe deposition in the visceral organs was found [25]. Overall, Fe SA is a biocompatible implant and shows better new mineralized bone growth around the implant.

In this study, a mild elevation in serum ALT was noted. The biocompatibility of the Fe SA was confirmed in a previous study of Fe SA implantation in rabbit tibia for three months [25], in which the histopathology of Prussian blue staining of the liver showed that no Fe was detected in the Fe SA group. Additionally, the percentage of Fe stores in the spleen in the Fe SA group showed no significant difference from those in the polymer SA group. In this study, some degradation products surrounding the Fe SA were found. A longer-term follow-up study is needed to confirm any occurrence of local inflammation reactions after further degradation of the Fe SA.

This study showed a similar short-term ultimate pullout strength between Ti and Fe SAs. Because of the structural difference, the stress concentration during the ultimate pullout test was located around the proximal site in the Ti SAs and around the distal site in the Fe SAs. A biomechanical study conducted in a polyurethane foam block and porcine bone showed a similar ultimate pullout strength and cyclic loading test results between open-construct coil-type PEEK SAs and Ti non-vented screw-type SAs [48]. The authors cannot attribute this to the superior biomaterial properties of Ti or Fe or the structural design of either the non-vented screw-type or open-construct coil-type SAs. Future studies comparing the same open-construct coil-type design with different biomaterials may determine whether nondegradable Ti or biodegradable Fe is superior for use in SAs.

3D printing technology allows for the rapid fabrication of porous implants and composite scaffolds which can be tailored to different orthopedic applications [25,49,50]. The Fe SA used in this study was produced by laser sintering using high purity biodegradable spherical Fe powder. 3D printing selective laser sintering technology allowed the accurate construction of the two-helical structure and the incorporation of porous structures in the thread, which could facilitate bone ingrowth to stabilize the anchor. Overall, the 3D printed open-construct biodegradable Fe SA showed good initial mechanical pullout strength, mineralized bone growth, and optimal gradual degradation.

This study had some limitations that should be addressed. First, the rabbit SST tear study analyzed the osteointegration between the bone and SA using histological evidence and micro-CT results. The ultimate pullout strength of the SST was evaluated. However, whether the occurrence of local inflammation reactions during Fe degradation may influence the SST healing requires further study. Second, this study showed that biodegradable Fe SA promoted better bone growth than commercialized Ti SAs. However, the two SAs in the experimental and control groups had different material properties and structural designs. Further studies using the same SA design with different materials are needed to clarify which material (Fe, Ti, or PEEK) is superior.

## 5. Conclusions

The in vitro test confirmed that the static biodegradable property of Fe SAs was 0.049 ± 0.002 mm/year and the pullout strength was similar to that of the Ti SAs. The micro-CT and histology confirmed that the Fe SAs exhibited better mineralized bone growth around the Fe SA as compared to the Ti SA in the rabbit rotator cuff tear model. Overall, the open-construct and double-helical Fe SAs produced using 3D printing technology could outperform the currently used Ti SAs in terms of their biomechanical properties and osteointegration capacity. Future research should focus on the biocompatibility of the long-term implantation of Fe SAs and the in vivo degradation rate of the biodegradable Fe SAs.

## Figures and Tables

**Table 1:** Blood biochemistry analysis.

	Week 0	Week 2	Week 6
BUN (mg/dL)	19.8 ± 3.19	18.43 ± 3.06	18.98 ± 2.83
ALT (U/L)	39.35 ± 13.33	51.95 ± 16.39	65.50 ± 23.19
Alb (g/dL)	4.12 ± 0.19	3.81 ± 0.21	4.07 ± 0.25
Cr (mg/dL)	1.35 ± 0.18	1.06 ± 0.17	1.19 ± 0.11

## Data Availability

The data used in this study are available in the main text of this article.

## References

[1] Tashjian R.Z. (2012). Epidemiology, natural history, and indications for treatment of rotator cuff tears. Clin. Sports Med..

[2] Kim J.H., Kim Y.S., Park I., Lee H.J., Han S.Y., Jung S., Shin S.J. (2020). A Comparison of open-construct PEEK suture anchor and non-vented biocomposite suture anchor in arthroscopic rotator cuff repair: A prospective randomized clinical trial. Arthroscopy.

[3] Longo U.G., Petrillo S., Loppini M., Candela V., Rizzello G., Maffulli N., Denaro V. (2019). Metallic versus biodegradable suture anchors for rotator cuff repair: A case control study. BMC Musculoskelet. Disord..

[4] Suchenski M., McCarthy M.B., Chowaniec D., Hansen D., McKinnon W., Apostolakos J., Arciero R., Mazzocca A.D. (2010). Material properties and composition of soft-tissue fixation. Arthroscopy.

[5] Stoetzel S., Malhan D., Wild U., Helbing C., Hassan F., Attia S., Jandt K.D., Heiss C., El Khassawna T. (2021). Osteocytes influence on bone matrix integrity affects biomechanical competence at bone-implant interface of bioactive-coated titanium implants in rat tibiae. Int. J. Mol. Sci.

[6] Schroder F.F., Huis In’t Veld R., den Otter L.A., van Raak S.M., Ten Haken B., Vochteloo A.J.H. (2018). Metal artefacts severely hamper magnetic resonance imaging of the rotator cuff tendons after rotator cuff repair with titanium suture anchors. Shoulder Elb..

[7] Micic I., Kholinne E., Kwak J.M., Koh K.H., Jeon I.H. (2019). Osteolysis is observed around both bioabsorbable and nonabsorbable anchors on serial magnetic resonance images of patients undergoing arthroscopic rotator cuff repair. Acta Orthop. Traumatol. Turc..

[8] Scholten D.J., Waterman B.R. (2020). Editorial commentary: Taking a "PEEK" at suture anchor composition following arthroscopic rotator cuff repair: Is bio really better?. Arthroscopy.

[9] Cho C.H., Bae K.C., Kim D.H. (2021). Biomaterials used for suture anchors in orthopedic surgery. Clin. Orthop. Surg..

[10] Qin Y., Yang H., Liu A., Dai J., Wen P., Zheng Y., Tian Y., Li S., Wang X. (2022). Processing optimization, mechanical properties, corrosion behavior and cytocompatibility of additively manufactured Zn-0.7Li biodegradable metals. Acta Biomater..

[11] Agarwal S., Curtin J., Duffy B., Jaiswal S. (2016). Biodegradable magnesium alloys for orthopaedic applications: A review on corrosion, biocompatibility and surface modifications. Mater. Sci. Eng. C Mater. Biol. Appl..

[12] Su T.-Y., Tang H.-Y., Jang J.S.-C., Chen C.-H., Chen H.-H. (2021). Design and development of magnesium-based suture anchor for rotator cuff repair using finite element analysis and in vitro testing. Appl. Sci..

[13] Putra N.E., Leeflang M.A., Minneboo M., Taheri P., Fratila-Apachitei L.E., Mol J.M.C., Zhou J., Zadpoor A.A. (2021). Extrusion-based 3D printed biodegradable porous iron. Acta Biomater..

[14] Venezuela J., Dargusch M.S. (2019). The influence of alloying and fabrication techniques on the mechanical properties, biodegradability and biocompatibility of zinc: A comprehensive review. Acta Biomater..

[15] Hermawan H. (2018). Updates on the research and development of absorbable metals for biomedical applications. Prog. Biomater..

[16] Zhang J., Hiromoto S., Yamazaki T., Huang H., Jia G.Z., Li H.Y., Yuan G.Y. (2017). Macrophage phagocytosis of biomedical Mg alloy degradation products prepared by electrochemical method. Mater. Sci. Eng. C Mater. Biol. Appl..

[17] Sanchez A.H.M., Luthringer B.J.C., Feyerabend F., Willumeit R. (2015). Mg and Mg alloys: How comparable are in vitro and in vivo corrosion rates? A review. Acta Biomater..

[18] Johnston S., Dargusch M., Atrens A. (2018). Building towards a standardised approach to biocorrosion studies: A review of factors influencing Mg corrosion in vitro pertinent to in vivo corrosion. Sci. China Mater..

[19] Shuai C.J., Li S., Peng S.P., Feng P., Lai Y.X., Gao C.D. (2019). Biodegradable metallic bone implants. Mat. Chem. Front..

[20] Gorejova R., Orinakova R., Kralova Z.O., Balaz M., Kupkova M., Hrubovcakova M., Haverova L., Dzupon M., Orinak A., Kal’avsky F. (2020). In vitro corrosion behavior of biodegradable iron foams with polymeric coating. Materials.

[21] Bowen P.K., Drelich J., Goldman J. (2013). Zinc exhibits ideal physiological corrosion behavior for bioabsorbable stents. Adv. Mater..

[22] Hagelstein S., Zankovic S., Kovacs A., Barkhoff R., Seidenstuecker M. (2022). Mechanical analysis and corrosion analysis of zinc alloys for bioabsorbable implants for osteosynthesis. Materials.

[23] Kraus T., Moszner F., Fischerauer S., Fiedler M., Martinelli E., Eichler J., Witte F., Willbold E., Schinhammer M., Meischel M. (2014). Biodegradable Fe-based alloys for use in osteosynthesis: Outcome of an in vivo study after 52 weeks. Acta Biomater..

[24] Hong D.H., Chou D.T., Velikokhatnyi O.I., Roy A., Lee B., Swink I., Issaev I., Kuhn H.A., Kumta P.N. (2016). Binder-jetting 3D printing and alloy development of new biodegradable Fe-Mn-Ca/Mg alloys. Acta Biomater..

[25] Tai C.-C., Lo H.-L., Liaw C.-K., Huang Y.-M., Huang Y.-H., Yang K.-Y., Huang C.-C., Huang S.-I., Shen H.-H., Lin T.-H. (2021). Biocompatibility and Biological performance evaluation of additive-manufactured bioabsorbable iron-based porous suture anchor in a rabbit model. Int. J. Mol. Sci..

[26] Md Yusop A.H., Ulum M.F., Al Sakkaf A., Hartanto D., Nur H. (2021). Insight into the bioabsorption of Fe-based materials and their current developments in bone applications. Biotechnol. J..

[27] Mathewson M.A., Kwan A., Eng C.M., Lieber R.L., Ward S.R. (2014). Comparison of rotator cuff muscle architecture between humans and other selected vertebrate species. J. Exp. Biol..

[28] Barber F.A., Herbert M.A., Richards D.P. (2003). Sutures and suture anchors: Update 2003. Arthroscopy.

[29] (2004). Standard Practice for Laboratory Immersion Corrosion Testing of Metals..

[30] Louati H., Uhthoff H.K., Culliton K., Laneuville O., Lapner P., Trudel G. (2018). Supraspinatus tendon repair using anchors: A biomechanical evaluation in the rabbit. J. Orthop Surg. Res..

[31] Chiu Y.R., Hsu Y.T., Wu C.Y., Lin T.H., Yang Y.Z., Chen H.Y. (2020). Fabrication of asymmetrical and gradient hierarchy structures of poly-p-xylylenes on multiscale regimes based on a vapor-phase sublimation and deposition process. Chem. Mat..

[32] Pyka G., Kerckhofs G., Schrooten J., Wevers M. (2014). The effect of spatial micro-CT image resolution and surface complexity on the morphological 3D analysis of open porous structures. Mater. Charact..

[33] Dalen G., Koster M. 2D & 3D particle size analysis of micro-CT images. Proceedings of the Bruker micro-CT User Meeting.

[34] Diekmann J., Bauer S., Weizbauer A., Willbold E., Windhagen H., Helmecke P., Lucas A., Reifenrath J., Nolte I., Ezechieli M. (2016). Examination of a biodegradable magnesium screw for the reconstruction of the anterior cruciate ligament: A pilot in vivo study in rabbits. Mater. Sci. Eng. C Mater. Biol. Appl..

[35] Wen C.Y., Qin L., Lee K.M., Chan K.M. (2009). Peri-graft bone mass and connectivity as predictors for the strength of tendon-to-bone attachment after anterior cruciate ligament reconstruction. Bone.

[36] Brooks B.D., Sinclair K.D., Grainger D.W., Brooks A.E. (2015). A resorbable antibiotic-eluting polymer composite bone void filler for perioperative infection prevention in a rabbit radial defect model. PLoS ONE.

[37] ISO, International Organization for Standardization (2012). 15189 Medical Laboratories-Requirements for Quality and Competence.

[38] Moravej M., Purnama A., Fiset M., Couet J., Mantovani D. (2010). Electroformed pure iron as a new biomaterial for degradable stents: In vitro degradation and preliminary cell viability studies. Acta Biomater..

[39] Obayi C.S., Tolouei R., Paternoster C., Turgeon S., Okorie B.A., Obikwelu D.O., Cassar G., Buhagiar J., Mantovani D. (2015). Influence of cross-rolling on the micro-texture and biodegradation of pure iron as biodegradable material for medical implants. Acta Biomater..

[40] Shuai C., Li S., Wang G., Yang Y., Peng S., Gao C. (2019). Strong corrosion induced by carbon nanotubes to accelerate Fe biodegradation. Mater. Sci. Eng. C Mater. Biol. Appl..

[41] Walker J., Shadanbaz S., Kirkland N.T., Stace E., Woodfield T., Staiger M.P., Dias G.J. (2012). Magnesium alloys: Predicting in vivo corrosion with in vitro immersion testing. J. Biomed. Mater. Res. B Appl. Biomater..

[42] Zainal Abidin N.I., Rolfe B., Owen H., Malisano J., Martin D., Hofstetter J., Uggowitzer P.J., Atrens A. (2013). The in vivo and in vitro corrosion of high-purity magnesium and magnesium alloys WZ21 and AZ91. Corros. Sci..

[43] Yang H., Jia B., Zhang Z., Qu X., Li G., Lin W., Zhu D., Dai K., Zheng Y. (2020). Alloying design of biodegradable zinc as promising bone implants for load-bearing applications. Nat. Commun..

[44] Mostaed E., Sikora-Jasinska M., Mostaed A., Loffredo S., Demir A.G., Previtali B., Mantovani D., Beanland R., Vedani M. (2016). Novel Zn-based alloys for biodegradable stent applications: Design, development and in vitro degradation. J. Mech. Behav. Biomed. Mater..

[45] Vojtěch D., Kubásek J., Šerák J., Novák P. (2011). Mechanical and corrosion properties of newly developed biodegradable Zn-based alloys for bone fixation. Acta Biomater..

[46] Chaler J., Louati H., Uhthoff H.K., Trudel G. (2020). Supraspinatus tendon transosseous vs anchor repair surgery: A comparative study of mechanical recovery in the rabbit. J. Orthop. Surg. Res..

[47] Bouxsein M.L., Boyd S.K., Christiansen B.A., Guldberg R.E., Jepsen K.J., Muller R. (2010). Guidelines for assessment of bone microstructure in rodents using micro-computed tomography. J. Bone Miner. Res..

[48] Kawakami J., Yamamoto N., Nagamoto H., Itoi E. (2018). Minimum distance of suture anchors used for rotator cuff repair without decreasing the pullout strength: A biomechanical study. Arthroscopy.

[49] Hsieh Y.-Y., Wu L.-C., Tsuang F.-Y., Chen C.-H., Chiang C.-J. (2021). Pull-Out capability of a 3D printed threadless suture anchor with rectangular cross-section: A biomechanical study. Appl. Sci..

[50] Anandhapadman A., Venkateswaran A., Jayaraman H., Ghone N.V. (2022). Advances in 3D printing of composite scaffolds for the repairment of bone tissue associated defects. Biotechnol. Prog..

