# Correction: Liu et al. 3D-Printed Double-Helical Biodegradable Iron Suture Anchor: A Rabbit Rotator Cuff Tear Model *Materials* 2022, *15*, 2801

**DOI:** 10.3390/ma15207226

**Published:** 2022-10-17

**Authors:** Wen-Chih Liu, Chih-Hau Chang, Chung-Hwan Chen, Chun-Kuan Lu, Chun-Hsien Ma, Shin-I Huang, Wei-Lun Fan, Hsin-Hsin Shen, Pei-I Tsai, Kuo-Yi Yang, Yin-Chih Fu

**Affiliations:** 1 Ph.D. Program in Biomedical Engineering, College of Medicine, Kaohsiung Medical University, Kaohsiung 80756, Taiwan; 2 Department Orthopedics, Kaohsiung Medical University Hospital, Kaohsiung Medical University, Kaohsiung 80756, Taiwan; 3 Regeneration Medicine and Cell Therapy Research Center, Kaohsiung Medical University, Kaohsiung 80756, Taiwan; 4 Orthopedic Research Center, Kaohsiung Medical University, Kaohsiung 80708, Taiwan; 5 Division of Plastic Surgery, Department of Surgery, Kaohsiung Medical University Hospital, Kaohsiung Medical University, Kaohsiung 80756, Taiwan; 6 Graduate Institute of Animal Vaccine Technology, College of Veterinary Medicine, National Pingtung University of Science and Technology, Pingtung 912301, Taiwan; 7 Department of Orthopedic Surgery, Kaohsiung Municipal Ta-Tung Hospital, Kaohsiung 80145, Taiwan; 8 Department of Healthcare Administration and Medical Informatics, Kaohsiung Medical University, Kaohsiung 80708, Taiwan; 9 Department of Orthopedics, College of Medicine, Kaohsiung Medical University, Kaohsiung 80708, Taiwan; 10 Institute of Medical Science and Technology, National Sun Yat-sen University, Kaohsiung 80420, Taiwan; 11 Department of Orthopedic Surgery, Park One International Hospital, Kaohsiung 81367, Taiwan; 12 Biomedical Technology and Device Research Laboratories, Industrial Technology Research Institute, Hsinchu 31057, Taiwan

In the original publication [1], there was a mistake in Figure 10. The authors apologize for any inconvenience caused and state that the scientific conclusions are unaffected. The correction was approved by the Academic Editor. The original publication has also been updated. Although there is no error in the figure legend, Figure 10A was the duplicate of Figure 10B, as follows:

**Figure 10:**
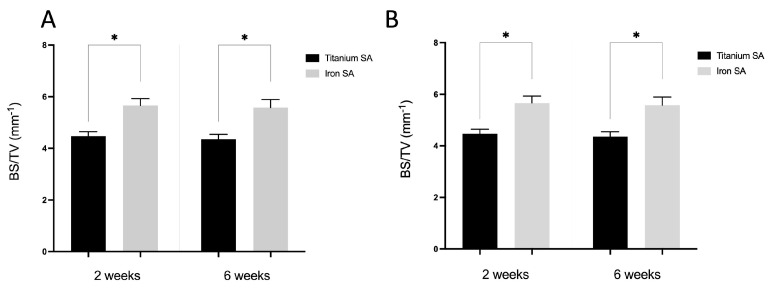
Micro-computed tomography (micro-CT) analysis. Quantitative evaluation of the bone volume (BV) between the bone and SAs. The tissue volume (TV, mm^3^), BV (mm^3^), and BS (mm^2^) were examined in a region of interest (ROI) of 200–1000 μm around the implant. (**A**) BV fraction (BV/TV, %) and (**B**) BS density (BS/TV, mm^−1^) represent the BV rate and bone tissue surface rate, respectively. Mean ± SEM. * *p* < 0.05.

The corrected Figure 10 should be:

**Figure 10:**
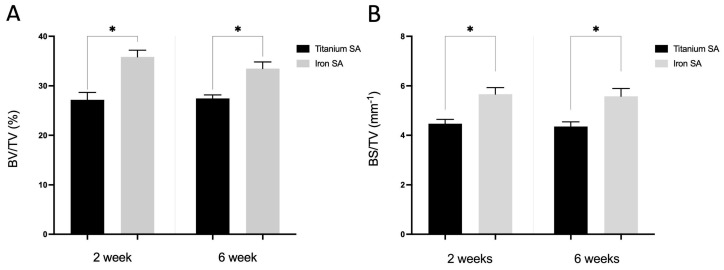
Micro-computed tomography (micro-CT) analysis. Quantitative evaluation of the bone volume (BV) between the bone and SAs. The tissue volume (TV, mm^3^), BV (mm^3^), and BS (mm^2^) were examined in a region of interest (ROI) of 200–1000 μm around the implant. (**A**) BV fraction (BV/TV, %) and (**B**) BS density (BS/TV, mm^−1^) represent the BV rate and bone tissue surface rate, respectively. Mean ± SEM. * *p* < 0.05.
